# High-flow nasal cannula oxygen therapy versus noninvasive ventilation for patients with blunt chest trauma: protocol for a randomized controlled trial

**DOI:** 10.1186/s13063-022-06507-2

**Published:** 2022-07-19

**Authors:** Qingcheng Zhu, Bingxia Wang, Yujie Li, Bingyu Ling, Jun Xu, Kui Jin, Ming Sun, Jianjun Zhu, Joseph Walline, Yunyun Wang, Peng Cao, Xiaojuan Guo, Dingyu Tan

**Affiliations:** 1 Department of Emergency Medicine, Clinical Medical College of Yangzhou University, Northern Jiangsu People’s Hospital, Yangzhou, 225001 China; 2 Department of Emergency Medicine, Peking Union Medical College Hospital, Chinese Academy of Medical Sciences, Beijing, 100730 China; 3 Department of Emergency Medicine, The First Affiliated Hospital of University of Science and Technology of China, Hefei, 230001 China; 4 Department of Emergency Medicine, Suqian People’s Hospital of Nanjing Drum-Tower Hospital Group, Suqian, 223800 China; 5 Emergency of Emergency Medicine, The Second Affiliated Hospital of Soochow University, Beijing, 215004 China; 6 Accident and Emergency Medicine Academic Unit, Prince of Wales Hospital, The Chinese University of Hong Kong, Hong Kong, China

**Keywords:** Acute respiratory failure, Blunt chest trauma, High-flow nasal cannula therapy, Noninvasive ventilation, Respiratory insufficiency

## Abstract

**Background:**

High-flow nasal cannula oxygen therapy (HFNC) is recommended by some scholars as an optimized respiratory support method for blunt chest trauma (BCT) patients. The basis of this recommendation is limited, however, and the efficacy of HFNC or noninvasive ventilation (NIV) in BCT patients has not yet been rigorously explored. This study aims to determine if HFNC is non-inferior to NIV in reducing treatment failure in moderate to severe BCT patients with acute respiratory failure.

**Methods:**

This will be a prospective, open-label, multicenter, non-inferiority, randomized controlled trial. Moderate to severe BCT patients with acute respiratory failure (100mmHg < PaO_2_/FiO_2_ ≦ 200mmHg) who do not need immediate intubation will be randomized to HFNC or NIV within 48 h after trauma. The primary outcome is treatment failure, defined as invasive ventilation or a switch in respiratory support modality (from HFNC to NIV or vice-versa). Secondary outcomes include arterial blood gas analysis and vital signs at 2 and 12 h after initiating HFNC or NIV treatment, as well as patients’ comfort scores, dyspnea scores, daily number of nursing airway care interventions, incidence of pneumonia or pneumothorax, facial skin breakdown, duration of NIV or HFNC, 28-day mortality, and total ICU and hospital lengths of stay. Based on an *α* error of 5% and a *β* error of 80%, with a non-inferiority limit of 9%, a sample size of 562 will be required to accomplish the trial goal, considering potential patient dropouts and nonparametric analysis.

**Discussion:**

We hypothesize that HFNC will be non-inferior to NIV in reducing treatment failure in moderate to severe BCT with acute respiratory failure. The results should be useful for judging whether HFNC could be an effective alternative to NIV to treat moderate to severe BCT patients, especially for those who do not tolerate or have contraindications for NIV.

**Trial registration:**

Chinese Clinical Trial Registry ChiCTR1800017313. Registered on July 24, 2018.

**Supplementary Information:**

The online version contains supplementary material available at 10.1186/s13063-022-06507-2.

## Background

Blunt chest trauma (BCT) is a common traumatic injury, especially common in motor vehicle collisions or falls. Mortality due to BCT can be as high as 36% and is the second-leading cause of death in trauma after head injuries [1]. Acute respiratory failure is an important cause of death in patients with BCT. Fifty to 70% of chest trauma is accompanied by acute respiratory failure, and up to 20% develop acute respiratory distress syndrome [2, 3].

Invasive mechanical ventilation is recommended for severe BCT patients with decreased levels of consciousness, significant respiratory distress, airway obstruction, severe hypoxemia, or hemorrhagic shock. However, for moderate to severe BCT patients not accompanied by the above-mentioned conditions, invasive ventilation may increase the incidence of hospital-acquired pneumonia and prolong the duration of mechanical ventilation and intensive care unit (ICU) length of stay, all of which can lead to worse outcomes [4].

In recent years, several studies have found that noninvasive ventilation (NIV) had a beneficial effect on BCT patients with acute respiratory failure. Two randomized controlled studies found that for flail chest or multiple rib fractures with respiratory failure, NIV reduced mortality and the incidence of pneumonia by 28% and 34%, respectively, compared with invasive mechanical ventilation [5, 6]. In a randomized controlled trial comparing NIV to standard oxygen therapy in patients with a ratio of PaO_2_ to the fraction of inspired oxygen (FiO_2_) [PaO_2_/FiO_2_] less than 200 mmHg within 48 h after BCT, early continuous use of NIV significantly reduced the rate of intubation compared to standard oxygen therapy alone (12% vs 40%) and shortened their hospital stay by as much as 7 days [7].

A meta-analysis also showed that for BCT patients with intact neurological function, stable hemodynamics, and no obvious respiratory distress, early NIV could significantly reduce the rate of endotracheal intubation and complications, as well as shorten the hospital length of stay, with a NIV failure rate of only 12–18% [8]. However, patients with BCT often have facial injuries, which is a contraindication to NIV. In addition, about 15% of patients cannot tolerate NIV [9]. NIV intolerance may lead to increased rates of treatment failure and affect the overall clinical outcome.

Recently, Kourouche proposed a concept of the “ChIP” bundle treatment protocol for BCT, which involved respiratory support, analgesic medications, preventative therapies, and surgical treatments [10]. In this protocol, high-flow nasal cannula oxygen therapy (HFNC) is used, which is an emerging type of respiratory support system which can provide high-flow mixed gases through special nasal cannulas with higher temperature and humidity than standard nasal cannulas. HFNC is the preferred method of respiratory support in the ChIP bundle. It was reported that the application of the ChIP protocol reduced the incidence of pneumonia in patients with BCT by 56% [11]. However, no research to date has confirmed the contribution of HFNC to this protocol.

Up to now, there have been only two small retrospective studies exploring the application of HFNC in BCT. One study showed that 18% of 105 patients with moderate to severe BCT [Abbreviated Injury Scale (AIS) chest score ≥ 3] received endotracheal intubation due to treatment failure with HFNC [12]. This rate is similar to the 12–18% NIV failure rate previously reported in the literature [8]. In another retrospective study, compared with standard oxygen therapy, HFNC significantly reduced both the intubation rate and the ICU length of stay in patients with moderate to severe BCT [13]. However, to the best of our knowledge, there are no published studies directly comparing the efficacy of HFNC to NIV in patients with BCT. The present protocol is conceived to test the hypothesis that HFNC is non-inferior to NIV in reducing treatment failure in moderate to severe BCT patients with acute respiratory failure.

## Methods

### Design and setting

This study is a prospective, open-label, multi-center, non-inferiority, randomized controlled trial. It will be carried out in the trauma ICUs of five tertiary university teaching hospitals in China: Northern Jiangsu People’s Hospital (Yangzhou), Peking Union Medical College Hospital (Beijing), The First Affiliated Hospital of University of Science and Technology of China (Hefei), Suqian People’s Hospital of Nanjing Drum-Tower Hospital Group (Suqian), and The Second Affiliated Hospital of Soochow University (Suzhou).

We designed the protocol according to the Standard Protocol Items: Recommendations for Interventional Trials (SPIRIT) statement. This study was prospectively registered at Chinese Clinical Trial Registry on July 24, 2018 (ChiCTR1800017313). Patients are expected to be enrolled during a 36-month period starting in January 2019. A web-based randomization schedule will be used with a block size of 10, and patients will be allocated according to a ratio of 1:1 to the NIV or HFNC groups. Attending physicians and study participants cannot be blinded to their grouping since the devices are clearly different. However, the investigators will be excluded from clinical decisions and will be blinded to the patient group allocations.

### Study population

Adult patients with moderate to severe BCT (AIS chest score ≥ 3) admitted within 48 h after a traumatic injury will be eligible if moderate hypoxemia (100mmHg < PaO_2_/FiO_2_ ≦ 200mmHg) developed while receiving standard oxygen therapy (mask ≥6 L/min). AIS chest score will be performed according to the method described by Baker et al. [14].

**Fig. 1:**
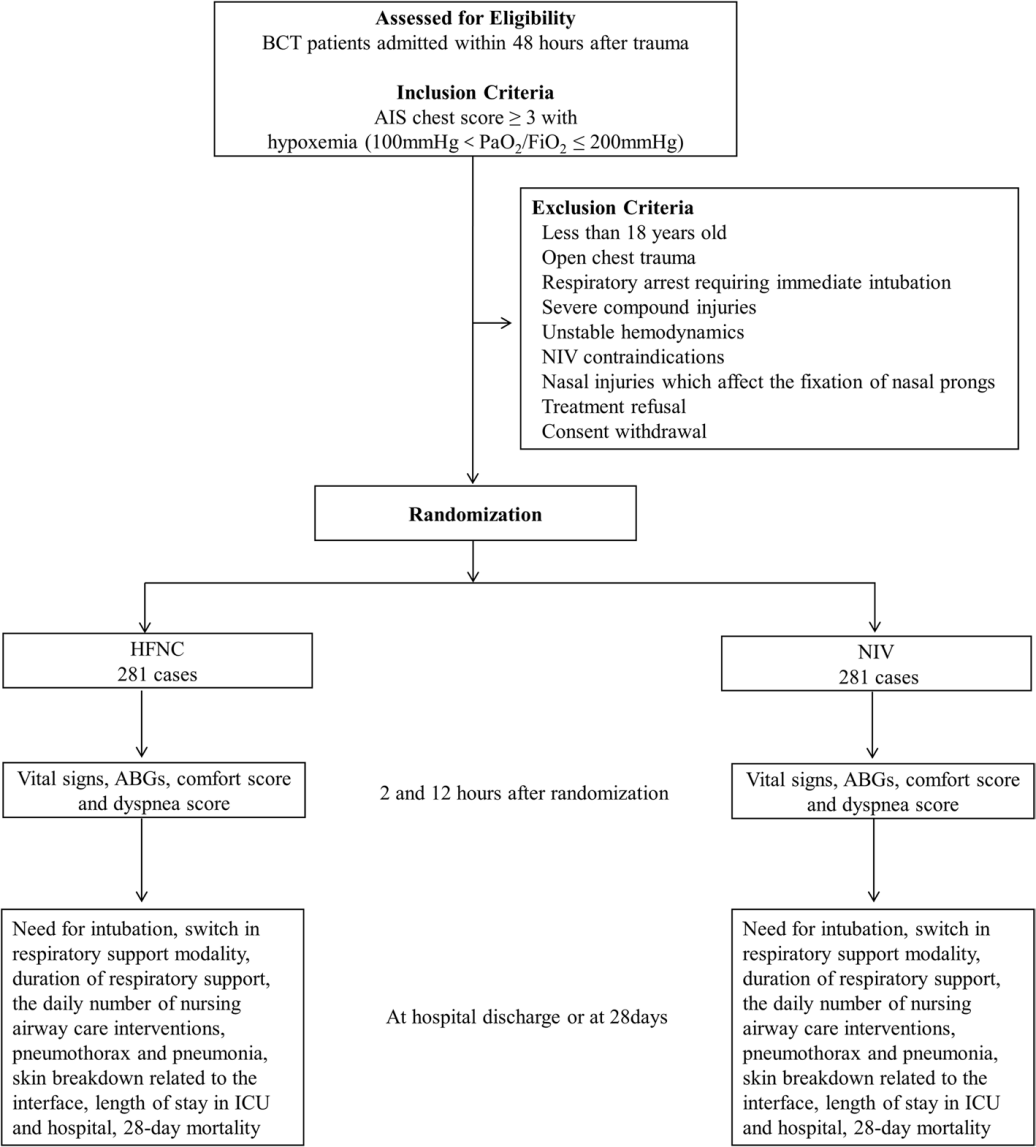
Study flow chart according to CONsolidated Standards of Reporting Trials. BCT, blunt chest trauma; AIS, Abbreviated Injury Scale; PO_2_, partial pressure of oxygen; FiO_2_, fraction of inspiration oxygen; ABG, arterial blood gas; ICU, intensive care unit; HFNC, high-flow nasal cannula oxygen therapy; NIV, noninvasive ventilation

Patients in one or more of the following criteria will be excluded (see Fig. 1): less than 18 years old, open chest trauma, those requiring immediate endotracheal intubation (for example, if a patient’s respiratory rate is > 40 times per minute or in respiratory arrest), severe injuries such as ruptured abdominal organs, decreased consciousness (severe brain injuries, Glasgow score <11), unstable hemodynamics (requiring dopamine > 8 μg/kg/min or norepinephrine > 0.2 μg/kg/min), need for urgent surgery, NIV contraindications (oral and facial trauma, poor sputum clearance capacity, tracheotomy, vomiting or aspiration), or nasal injuries affecting the insertion or stability of nasal prongs (such as nasal bone fractures or significant nasal epistaxis).

Eligible patients will be recruited by investigators (attending physicians) in each study hospital center. After participant selection, physicians will guide patients or their next of kin through informed consent (or refusal), which includes a brief introduction of the study, interventions, and the contact information of the investigators. A research assistant in each study center is then notified about patients who are willing to participate in this trial. Two copies of the informed consent are kept, one with the participants and one with the research team. The consent form and information materials are available from the corresponding author on request.

### Study groups

Within 3 h of meeting inclusion criteria and obtaining informed consent, patients will be randomly allocated to receive either NIV or HFNC. Patients randomized to the HFNC group will receive HFNC (AIRVO™ 2, Fisher & Paykel Healthcare, Auckland, New Zealand) using suitable large-bore nasal prongs selected according to the size of the patients’ nostrils. The initial airflow will be set at 50 L/min and adjusted according to patient tolerance. To reach an absolute humidity of 44mg H_2_O/L, the temperature will be set to 37°C. FiO_2_ will be initially set at 1.0 and adjusted to maintain a SpO_2_ ≥ 92%. HFNC will be continuous if it is well tolerated, or it can be used intermittently according to clinician decision. During the termination of HFNC, standard oxygen therapy will be used to maintain a SPO_2_ ≥ 92%.

Subjects randomized to the NIV group will receive NIV (BiPap Vision or PHILIPS V60) in spontaneous or timed (S/T) mode using a standard oral-nasal (full-face) mask (RT040). The parameters will be adjusted adaptively: the expiratory positive airway pressure (EPAP) settings will be increased gradually from 4 cmH_2_O to make sure the patient can trigger the ventilator with each inhalation. The inspiratory positive airway pressure (IPAP) will be initially set to 8 cmH_2_O and gradually increased to achieve a satisfactory tidal volume (8mL/kg) with acceptable tolerance. The pressure level and FiO_2_ will be adjusted to maintain a respiratory rate ≤ 28/min and a SpO_2_ ≥ 92%. The NIV should be applied as continuously as possible for 24 h. To improve tolerance, a short break from NIV will be allowed with the consent of the attending physician. Oxygen therapy during any interruption of NIV will be the same as for the HFNC group.

Patients in both groups will be in a semi-recumbent position unless contraindicated. A multi-modal pain control regimen will be adopted in all participants. Epidural analgesia with sufentanil plus bupivacaine will be preferred, combined with oral celecoxib (200 mg twice daily). When the visual analogue scale of pain is greater than 6, 100 mg tramadol will be given intravenously as a remedial treatment. For patients with contraindications to regional anesthesia (acute spine fracture, unstable pelvic fracture, coagulopathy, et al.), analgesia based on intravenous remifentanil will be administered, and the dosage will be determined using pain scales. In addition to respiratory support and pain management, other treatments will be supplied to both groups according to current guidelines, including hemostasis, external fixation of the chest wall, thoracentesis or thoracic closed drainage, fluid resuscitation, blood transfusion, or any surgical operation, if necessary [15].

### Outcomes

The primary outcome is treatment failure, defined as intubation and invasive ventilation, or a switch in respiratory support modality (i.e., changing from HFNC to NIV or vice-versa). Detailed information about treatment failure will be recorded from baseline to 28 days after randomization. Secondary outcomes include arterial blood gas (ABG) analysis results [pH, PaO_2_, PaCO_2_, and PaO_2_/FiO_2_] and vital signs [respiratory rate, heart rate, and blood pressure] at 2 and 12 h after initiating NIV or HFNC, as well as the patients’ comfort scores, dyspnea scores, the daily number of nursing airway care interventions, the incidence of pneumonia or pneumothorax, skin breakdown related to the airway device, duration of NIV or HFNC, total ICU and hospital lengths of stay, and 28-day mortality.

In the initial trial registry entry, we set arterial blood gas analysis results and vital signs as the primary outcomes. Considering that the primary purpose of this study was to compare the efficacy and safety of HFNC and NIV for BCT patients, we changed the primary endpoint to treatment failure before starting patient enrollment. The amendments were approved by the study’s steering committee and the ethics review committee of Northern Jiangsu People’s Hospital.

### Data collection and management

We will collect the personal characteristics of each BCT patient (age, gender, height, and weight), medical history, time of traumatic injury, mechanism of injury, injured organs, imaging results, injury severity score (ISS), AIS chest score, and Sequential Organ Failure Assessment (SOFA) score at enrollment. In addition, we will also collect vital signs (respiratory rate, heart rate, and blood pressure), comfort score, dyspnea score, and ABG results from just prior to initiating HFNC or NIV treatment after patient enrollment (see Table 1). Patients’ dyspnea will be evaluated using the Borg rating scale [16], and patients’ comfort score will be assessed using a 10-cm visual analogue scale, in which 1 means no discomfort and 10 means maximal discomfort [17].

**Table 1:** Schedule of enrolment, interventions, and assessments

	Study period					
	Enrolment	Allocation	Post-allocation			Close-out
Timepoint	0	0	2 h	12 h	1–28 days maximum	28 days maximum
Enrolment						
Eligibility screen	×					
Informed consent	×					
Allocation		×				
Interventions						
NIV		×				
HFNC		×				
Assessments						
Baseline variables						
Demographics	×					
Medical history	×					
Mechanism of injury	×					
Injury severe score	×					
Chest AIS score	×					
SOFA score	×					
Outcome variables						
Intubation			×	×	×	
Treatment switch			×	×	×	
Vital signs		×	×	×	×	
Comfort score		×	×	×	×	
Dyspnea score		×	×	×	×	
Arterial blood gas		×	×	×	×	
Number of nursing airway care interventions					×	
Pneumonia or pneumothorax					×	
Skin breakdown					×	
Causes of treatment failure						×
NIV/HFNC duration						×
Hospital length of stay						×
ICU length of stay						×
28-day mortality						×

*HFNC* high-flow nasal cannula oxygen therapy, *NIV* noninvasive ventilation, *AIS* Abbreviated Injury Scale, *SOFA* Sequential Organ Failure Assessment, *ICU* intensive care unit

At 2 and 12 h after allocation, vital signs, ABG results, comfort score, and dyspnea score will be collected. We will then re-collect this information every day until ICU discharge. Moreover, we will separately record each patient’s HFNC or NIV settings, any episodes of skin breakdown related to the airway interface, the number of nursing airway care interventions, and any intolerance to HFNC or NIV due to claustrophobia, excessive air flow or pressure, breathlessness, noise, ocular irritation, headache, or other reasons.

Furthermore, we will also record any episodes of endotracheal intubation, or switching of respiratory support modality, including the reason and time for these events. We will also document any diagnosed episodes of pneumothorax or pneumonia, as well as each patients’ length of stay in the ICU and hospital, and their 28-day mortality.

Any decision to intubate a patient will be made by the patient’s attending physician. Criteria for intubation in this trial are cardiac arrest or apparent hemodynamic instability, significant hypoxemia (SpO_2_ < 85% while under sufficient oxygen therapy), respiratory acidosis with a pH < 7.25 under optimal medical management, severe disturbances of consciousness (Glasgow score <11), obvious aspiration or significantly reduced capacity for airway protection, active use of accessory muscles with thoraco-abdominal paradoxical movements, severe dyspnea (respiratory rate > 40 per minute), or respiratory depression (respiratory frequency < 8 per minute) [7].

All data will be collected on paper case report forms (CRFs) from patients’ electronic medical records. The data manager will collect CRFs and enter data to a dedicated web-based electronic database in de-identified form. The electronic data will be backed up, and the backed-up electronic data will be kept secure by the sponsor. The sponsor will check the data again according to the CRFs to ensure data accuracy. Treatment allocation will be anonymously processed to allow the independent statistician in this trial to be blinded. Only the trial investigators will have access to the final dataset.

The sponsor and statisticians will have access to the preliminary data set to enable needed statistical work. Doctors authorized by the sponsor will have access for data entry and for reviewing data at their site. Participants will be allocated an individual trial identification number and the allocation information will be stored in a separate, secure database. All data will be kept confidential and will not be disclosed. Anonymized trial data will be shared with other researchers upon reasonable request to contribute to future international prospective meta-analyses.

### Statistical analysis and sample-size calculation

Reported intubation rates in BCT patients receiving NIV range from 12 to 18% [8], so the sample size is estimated assuming a baseline failure rate (including modality switch) of 18–23% for each group and a predefined non-inferiority margin of 9% for HFNC. We set the non-inferiority margin according to relevant data [18, 19], and after discussion with three senior pulmonologists. We computed a sample size of 488 patients in this trial, using an *α* = 0.05 (one-sided) and *β* = 0.20, with a non-inferiority limit of 9%. After considering a potential patient dropouts and nonparametric analysis, 562 patients (281 per modality) will be the target sample size for this trial.

All statistical analyses will be performed on an intention-to-treat basis by an independent investigator who is blinded to treatment allocation. The Kolmogorov–Smirnov test will be used to test the normal distribution of data. Normally distributed data will be expressed as means ± standard deviation, while skewed distributed data will be reported as medians with interquartile (25th–75th) percentiles. The two modality groups will be compared using *t*-tests or Mann–Whitney *U*-tests. Numeric data will be presented as a percentage (%), using chi-squared or Fisher’s exact probability tests. Significance will be set at < 0.05 and all data analysis will be conducted with SPSS 26.0 (IBM Corporation, Armonk, NY, USA).

### Study organization

The steering committee of this trial consists of two principal investigators (XJG, DYT) plus four members with recognized expertise on HFNC/NIV in BCT patients. Each participating hospital has a local investigator in charge. To achieve consistency in this trial, study coordinators, site investigators, and all research staff involved in research implementation, data collection, and data recording from all sites will be centrally trained in the trial’s standard operating procedures and study protocol.

The data monitoring committee will be set up by Northern Jiangsu People’s Hospital. It will be composed of a group of independent experts independent from the sponsor and competing interests. The role of data monitoring committee is to check the quality and integrity of the study data in each research site and question any problematic data. Protocol compliance, participant recruitment, and data entry will also be supervised by the data monitoring committee every month.

The trial steering committee, the data monitoring committee, and the ethics committee will meet every 2 weeks online or onsite to ensure that the study is being conducted in accordance with the study protocol.

Northern Jiangsu People’s Hospital is the sponsor, which is responsible for the study design, collection, analysis, and interpretation of data and in writing the manuscript. Northern Jiangsu People’s Hospital has the final power to interpret these activities.

Formal protocol amendments will be approved by the trial steering committee and the ethics committee of Northern Jiangsu People’s Hospital prior to implementation. Patients will be provided revised informed consent forms to document any modifications. If the study protocol is to be amended, the primary investigator will inform the steering committee. The primary investigator will then add the amended protocol into the investigator site file. Any deviations from the protocol will be fully documented using a protocol breach reporting form.

### Strengths and limitations of this study

This will be the first randomized controlled trial to observe the effect of HFNC directly compared to NIV on BCT patients with acute respiratory failure. This study will investigate the ability of HFNC to prevent treatment failure in moderate to severe BCT patients with acute respiratory failure. This study will also explore the relative tolerance of BCT patients to either NIV or HFNC, as well as the frequency of switching between the two modalities. Blinding is not applicable due to the nature of the intervention.

## Discussion

Respiratory support is an important part of treating BCT. Standard oxygen therapy, NIV, and invasive mechanical ventilation are commonly used respiratory support methods in patients with BCT. For BCT patients at risk for acute respiratory failure who do not require early invasive ventilation, the ideal method of respiratory support is not yet clear. Although some studies have suggested NIV was effective for treating BCT, two recent guidelines did not make or made only low-grade recommendations for NIV in patients with BCT [20, 21]. Likewise, although there is some evidence showing that HFNC could be a useful alternative to NIV, there have been no randomized controlled trials to date to demonstrate this efficacy.

In addition to the contraindications to NIV due to nasal or facial trauma, poor patient-ventilator synchrony related to NIV intolerance is a common problem in the clinical application of NIV. In a recent study, 29% of NIV failures were attributed to treatment intolerance, which was significantly higher than the 4% treatment intolerance rate for HFNC [22]. The nasal prong design of HFNC does not lead to a sense of claustrophobia, enabling patients to eat, drink, and talk, resulting in increased compliance compared to NIV. The heating and humidifying function of HFNC enables the gas delivered to reach an absolute humidity of 44mg H_2_O/L and a temperature of 37°C, which effectively promotes the discharge of secretions while avoiding side effects such as dry mucous membranes [23]. Several studies have confirmed that the comfort and tolerance of HFNC is significantly better than NIV [17, 24, 25].

Pneumothorax is one of the most common NIV-related adverse events in patients with BCT, with an incidence of 5.5–24% [2]. NIV, a kind of positive pressure ventilation, may worsen mild pneumothorax in patients with BCT. Meanwhile, inappropriate positive airway pressures may increase the risk for an iatrogenic pneumothorax. HFNC provides a low level of positive airway pressure without pressure support in a more comfortable way, which may decrease the risk of pneumothorax in patients with BCT.

In a multicenter study of hypoxic respiratory failure, HFNC had a similar tracheal intubation rate compared to NIV, although HFNC had a significantly reduced the 90-day mortality rate [24]. Acute hypoxic respiratory failure is common in patients with BCT, so HFNC may theoretically have a good effect on BCT patients. HFNC was recommended by some scholars as an important part of respiratory management in BCT patients; however, the recommendation was only based on two small retrospective observational studies, and the specific therapeutic differences between HFNC and NIV were not explored. To the best of our knowledge, this is the first randomized controlled trial to compare the efficacy of HFNC and NIV on BCT patients with acute respiratory failure. Uniform criteria to initiate, stop, and resume HFNC or NIV have already been established and will be used to ensure that all patients receive similar treatment. Blinding in our study is not possible for both patients and clinicians since the devices are clearly different. However, investigators will be excluded from clinical decisions, and all analyses will be carried out in a blinded manner.

In conclusion, this study will test the hypothesis that HFNC is non-inferior to NIV in reducing treatment failure in moderate to severe BCT and acute respiratory failure. Results from this trial should be useful for optimizing respiratory support in future patients with moderate to severe BCT.

## Trial status

The trial is currently recruiting patients. The final study protocol (version 3.0) was set on August 28, 2018. The first participant was enrolled on January 13, 2019. Recruitment was originally expected to be completed by December 31, 2021. Due to the impact of COVID-19, the recruitment completion time was delayed and is currently expected to complete by June 30, 2022.

## Supplementary Information

**Additional file 1.**

## Data Availability

This trial does not involve collecting biological specimens for storage. The authors will share all the data and materials collected during the study, after de-identification upon reasonable request.

## Abbreviations

BCT: Blunt chest trauma
NIV: Noninvasive ventilation
ICU: Intensive care unit
AIS: Abbreviated Injury Scale
HFNC: High-flow nasal cannula oxygen therapy
PO_2_: Partial pressure of oxygen
FiO_2_: Fraction of inspiration oxygen
ABG: Arterial blood gas
SOFA: Sequential Organ Failure Assessment
